# Meta-analysis data for the literature on dividend smoothing

**DOI:** 10.1016/j.dib.2019.01.002

**Published:** 2019-01-06

**Authors:** Erik Fernau, Stefan Hirsch

**Affiliations:** a University of Mannheim, Finance Area, L 9, 1-2, Germany; b Technical University of Munich, School of Management, Germany

## Abstract

The dataset presented in this data article was compiled from 100 published and unpublished empirical studies that employed Lintner׳s dividend payout model or related extensions over the period 1957–2016. Besides the reported degree of dividend smoothing and its estimation precision the data include a wide set of underlying study design characteristics such as the time period of analysis, the type of firms investigated or the econometric estimator employed. The data are related to the research article “What drives dividend smoothing? A meta-regression analysis of the Lintner model” (Fernau and Hirsch, 2018).

**Specifications table**

**Table:** 

Subject area	*Economics*
More specific subject area	*Financial economics*
Type of data	*Table, excel file*
How data were acquired	*Survey of the literature on dividend smoothing based on the Linter model and extensions*
Data format	*Raw*
Experimental factors	*Dataset including information on study design and results from primary studies focusing on firm dividend smoothing between 1957–2016*
Experimental features	*Dataset comprises 998 dividend smoothing regression coefficients and underlying study design characteristics from 100 empirical studies*
Data source location	*Scientific literature on dividend smoothing published in EU, US, and developing countries*
Data accessibility	*With this article*
Related research article	*Fernau, E. and Hirsch, S. What drives dividend smoothing? A meta-regression analysis of the Lintner model. International Review of Financial Analysis,*https://doi.org/10.1016/j.irfa.2018.11.011[1].

**Value of the data**

• The dataset provides an extensive overview of the empirical literature on dividend smoothing.
• Researchers can gain holistic insights on dividend smoothing and the factors that drive its degree.
• The importance of research design characteristics for the resulting degree of dividend smoothing can be evaluated using this dataset.
• The dataset can serve to detect publication biases across the empirical literature on dividend smoothing.
• The dataset can provide a useful benchmark and/or starting point for meta-analysis based on further issues of payout policy such as stock repurchases.

## 1 Data

Our dataset comprises 998 estimated dividend smoothing coefficients derived from 100 published and unpublished empirical studies that employed Lintner׳s dividend payout model or related extensions over the period 1957–2016. For each dividend smoothing coefficient, the standard error is included as a measure of estimation precision and a wide set of underlying study design characteristics such as the type of publication, the econometric specification used to generate the coefficient as well as the analyzed period, sector and region are added. Table 1 provides an overview of study design characteristics of the empirical studies included in the dataset.

**Table 1:** Study design characteristics of studies included in the meta data on dividend smoothing.

Study ID	No. of years	Post 1998	Working paper	Econometric approaches	Control variables	Cash-flow	Countries	Industries
1	2	1	0	OLS	Ownership	0	Developing country	Service & consumer
2	14	0	0	OLS	No controls	1	Developing country	
3	6	1	1	OLS, GMM, FE, other methods	Size	0	Developing country	
4	6	1	0	OLS, GMM, FE, other methods	Debt, Ownership, Size, other controls	0	Developing country	
5	19	0	0	FE	Size	0	US	
6	10	1	0	OLS	Other controls	0	Other countries	Financial firms
7	6	1	0	OLS	Other controls	0	Developing country	
8	10	1	0	Other methods	Other controls	0	Other countries	
9	10	1	0	OLS, FE, other methods	Size	0	Developing country	
10	17	1	0	GMM, FE, other methods	Size	1	UK	
11	16	0	0	Other methods	No controls	0	Other countries	
12	16	0	0	Other methods	No controls	0	Other countries	Financial firms
13	8	1	1	GMM, FE	Size, other controls	0	Other countries	
14	11	1	0	OLS	No controls	0	Developing country	Manufacturing firms
15	11	1	0	OLS	No controls	0	Developing country	Financial firms
16	22	0	0	OLS, GMM, FE	Size	0	EU	
17	22	0	0	OLS, GMM	Ownership, other controls	1	EU	
18	21	1	0	OLS, GMM, FE	Size	0	EU	
19	11	1	0	OLS	Other controls	0	Developing country	
20	10	1	0	GMM	No controls	0	Other countries	Financial firms
21	14	0	0	GMM	Debt, other controls	0	other countries	
22	10	1	1	OLS	No controls	0	US	
23	7	1	0	OLS	No controls	0	Other countries	
24	5	1	0	OLS, FE	Size, other controls	0	Developing country other countries	Manufacturing firms
25	27	0	0	OLS	No controls	0	EU	Financial firms, Manufacturing firms
26	19	1	0	OLS	Other controls	0	Developing country	
27	5	1	1	OLS, FE	Size, other controls	0	UK	
28	13	0	1	Other methods	No controls	0	EU	
29	11	1	0	OLS	No controls	0	Developing country	Financial firms
30	9	1	0	OLS, GMM	Other controls	0	Developing country	
31	25	1	0	OLS, GMM, FE	Ownership, Size, other controls	0	EU	Manufacturing firms
32	41	0	0	OLS	No controls	0	US	
33	87	0	0	OLS	No controls	0	US	
34	19	0	0	OLS	No controls	0	US other countries	
35	6	1	0	OLS	Ownership	0	US	
36	10	0	0	Other methods	Other controls	0	EU	
37	26	0	0	OLS	Other controls	0	US	
38	22	0	0	Other methods	No controls	0	US	
39	16	0	0	OLS, FE, other methods	Size, other controls	0	US	
40	3	0	0	OLS	No controls	0	US	
41	8	0	0	GMM	Debt, other controls	0	Other countries	Financial firms
42	12	0	0	OLS, other methods	Other controls	0	UK	
43	133	0	0	OLS	No controls	0	Other countries	Financial firms
44	122	0	0	Other methods	No controls	0	US	
45	10	0	0	OLS, GMM, FE, other methods	Size	1	EU	
46	4	1	0	OLS	Debt, other controls	0	Other countries	
47	7	0	0	FE	Size	0	EU	
48	5	1	0	GMM	Debt, Ownership	0	Developing country	
49	7	1	0	OLS, GMM	Other controls	0	Developing country	
50	3	1	0	OLS	Debt, other controls	1	Developing country	Service & consumer, Manufacturing firms
51	12	0	0	OLS, other methods	Debt, other controls	0	UK	Financial firms
52	21	0	0	OLS, FE, other methods	Debt, Ownership, Size, other controls	0	US	
53	6	0	0	OLS	no controls	0	US	
54	17	1	0	FE, other methods	Ownership, Size, other controls	1	Other countries	
55	10	1	0	FE	Debt, Size	0	Developing country	Financial firms
56	16	1	0	GMM	Other controls	0	Developing country	
57	37	0	0	GMM	Other controls	0	Developing country	
58	6	1	0	OLS	No controls	0	Developing country	
59	3	0	0	FE, other methods	Ownership, Size	0	EU	
60	13	0	0	OLS, GMM	Debt, Ownership	0	UK	
61	11	1	0	OLS, FE, other methods	Size, other controls	0	Other countries	
62	5	1	1	OLS	Debt, other controls	0	Other countries	Financial firms
63	4	1	0	OLS, FE, other methods	Debt, Ownership, Size, other controls	0	EU	
64	7	0	0	OLS, FE	Debt, Ownership, Size, other controls	0	Developing country	
65	5	1	0	OLS	Other controls	1	Other countries	Service & consumer
66	2	0	0	OLS	Other controls	0	EU	
67	20	0	1	FE	Size	0	Other countries	
68	9	1	1	GMM, FE, other methods	Size, other controls	0	Developing country	
69	16	0	0	OLS	No controls	0	Developing country	
70	32	0	0	Other methods	Other controls	0	Developing country	
71	4	0	0	OLS, other methods	Other controls	0	US	
72	7	1	0	OLS, GMM, FE, other methods	Debt, Size, other controls	0	Developing country	
73	7	1	0	OLS, GMM, FE, other methods	Ownership, Size, other controls	0	Developing country	Financial firms
74	18	0	0	OLS	No controls	0	Other countries	
75	23	0	0	OLS	No controls	0	US	
76	15	0	0	OLS, FE, other methods	Size	0	Developing country	
77	2	1	0	OLS	Debt, other controls	0	Developing country	Financial firms
78	9	0	0	GMM	Other controls	0	Developing country	
79	8	0	0	FE	Size	0	Developing country	Service & consumer, Manufacturing firms
80	20	0	0	FE	Size, other controls	0	US	
81	7	1	0	FE	Size	0	EU	Service & consumer, Manufacturing firms
82	11	1	0	GMM	Debt, Ownership, other controls	0	EU	
83	70	0	1	OLS	No controls	0	US	
84	8	0	1	OLS	Ownership, other controls	0	other countries	
85	6	1	0	OLS	Debt, Ownership, other controls	0	Developing country	
86	9	1	0	GMM	Debt, Ownership, other controls	1	EU	
87	7	0	0	GMM	Debt, Ownership, other controls	0	UK	
88	27	0	0	OLS, other methods	Other controls	1	EU	
89	11	1	0	FE	Debt, Ownership, Size, other controls	0	Other countries	
90	5	0	0	OLS	Ownership	0	UK	
91	10	1	0	FE	Debt, Size, other controls	0	Developing country	Financial firms
92	11	1	0	OLS	Other controls	1	Developing country	Financial firms
93	2	0	0	OLS	Other controls	0	Other countries	Financial firms
94	6	1	0	FE	Size	0	Developing country	
95	7	0	0	GMM	Debt, Ownership, other controls	0	UK	
96	15	0	0	OLS	Other controls	0	US	
97	6	1	0	OLS, FE	Size	0	EU	
98	11	1	0	OLS, other methods	Debt, Ownership	0	Other countries	
99	9	1	0	FE, other methods	Size	0	Other countries	Manufacturing firms
100	7	1	0	OLS	Debt, Ownership	0	Developing country	Financial firms

Notes: A value of 1 for the characteristics “Post 1998”, “Working paper” and “Cash-Flow” indicates presence of the characteristic in the respective study.

## 2 Experimental design, materials and methods

This dataset was compiled based on a survey of the literature that focuses on the empirical estimation of Lintner׳s dividend payout model or related extensions. The literature search followed the Meta-Analysis of Economics Research Reporting Guidelines provided by [2]. Using these guidelines, we conducted the following steps to derive the final dataset. First, we screened the databases Econstor, Google Scholar, SSRN, Jstor, Wiley, Business Source Premier EBSCOhost, NBER, Econ papers using reasonable combinations of key terms related to Lintner׳s dividend payout model (“dividends”, “payouts”, “Lintner model”, “dividend smoothing”,”target payout”, and “speed of adjustment”). Second, we used snowballing techniques to search for publications that were not detected through the key term search. Third, we screened the resulting set of potentially relevant published and unpublished research papers to only include articles that conducted a quantitative empirical estimation of Lintner׳s dividend payout model or a related extension and that have not been published in “predatory journals” according to “Beall׳s List of Predatory Journals and Publishers”. Finally, all specifications derived from working paper versions of later published articles were excluded to avoid duplicative observations.

To ensure a consistent and uniform interpretation of dividend smoothing across the dataset we converted all speed of adjustment coefficients to dividend smoothing coefficients as defined in [3]. The final dataset comprises 100 studies on dividend smoothing that were published between 1957 and 2016 leading to 998 dividend smoothing coefficients. As a measure of precision with which the dividend smoothing coefficients have been estimated their standard errors are included. In cases where only *p*-, or *t*-values were reported those were converted to standard errors.

Each dividend smoothing coefficient relates to a unique combination of underlying study design characteristics. The dataset therefore also comprises an extensive set of variables that were selected to reflect in a best possible way the set of study design characteristics used to generate each smoothing coefficient. Table 1 depicts the study design characteristics by Study ID as included in the Excel file. These characteristics are described in the following. “No. of years” indicates the length of the time series dimension of the analyzed panel. “Post 1998” indicates whether the mean year of the analyzed sample is post 1998. 1998 was chosen to reflect that at the end of the 1990s significant levels of stock repurchases served as an additional payout option in a large number of countries covered by studies in our dataset. “Working paper” classifies articles as working papers if they have been finalized more than five years ago (i.e. pre 2012) and not been published since then [4]. The econometric approaches used to estimate the dividend smoothing coefficients are classified as OLS, GMM, fixed effects and other estimators, where the latter includes Probit, Tobit, Logit or Random Effects estimations. It is also captured whether a dividend smoothing coefficient was estimated based on an extension of the “classical” Linter model [3] that includes additional explanatory variables. Among those variables are the firms` level of debt, size, ownership structure or other firm-specific characteristics that due to their heterogeneity across literature are grouped as a joint set of ‘other’ explanatory variables. Moreover, “Cash-flow” indicates whether cash-flow was used as an alternative earnings measure. Finally, smoothing coefficients are classified based on the countries and industries in which the analyzed firms operate. In case of an industry focus of the specific study, we distinguish between the service and consumer goods, financial, and manufacturing sector. As regards countries we differentiate between the US, the UK, other EU countries, and developing countries (classified based on the definition of the World Bank) while “other countries” captures the remaining set of countries (e.g. Canada, China, or Russia) that given its heterogeneity is included in a single dummy variable.
